# An Active‐Matrix Synaptic Phototransistor Array for In‐Sensor Spectral Processing

**DOI:** 10.1002/advs.202406401

**Published:** 2024-08-21

**Authors:** Dingwei Li, Yitong Chen, Huihui Ren, Yingjie Tang, Siyu Zhang, Yan Wang, Lixiang Xing, Qi Huang, Lei Meng, Bowen Zhu

**Affiliations:** ^1^ Westlake Institute for Optoelectronics Hangzhou 311421 China; ^2^ Key Laboratory of 3D Micro/Nano Fabrication and Characterization of Zhejiang Province School of Engineering Westlake University Hangzhou 310024 China; ^3^ College of Information Science and Electronic Engineering Zhejiang University Hangzhou 310027 China; ^4^ Beijing National Laboratory for Molecular Sciences CAS Key Laboratory of Organic Solids Institute of Chemistry Chinese Academy of Sciences Beijing 100190 China; ^5^ Institute of Advanced Technology Westlake Institute for Advanced Study Hangzhou 310024 China

**Keywords:** active‐matrix array, bidirectional photoresponse, bulk heterojunction, optoelectronic synapse

## Abstract

The human retina perceives and preprocesses the spectral information of incident light, enabling fast image recognition and efficient chromatic adaptation. In comparison, it is reluctant to implement parallel spectral preprocessing and temporal information fusion in current complementary metal‐oxide‐semiconductor (CMOS) image sensors, requiring intricate circuitry, frequent data transmission, and color filters. Herein, an active‐matrix synaptic phototransistor array (AMSPA) is developed based on organic/inorganic semiconductor heterostructures. The AMSPA provides wavelength‐dependent, bidirectional photoresponses, enabling dynamic imaging and in‐sensor spectral preprocessing functions. Specifically, near‐infrared light induces inhibitory photoresponse while UV light results in exhibitory photoresponse. With rational structural design of the organic/inorganic hybrid heterostructures, the current dynamic range of phototransistor is improved to over 90 dB. Finally, a 32 × 64 AMSPA (128 pixels per inch) is demonstrated with one‐switch‐transistor and one‐synaptic phototransistor (1‐T‐1‐PT) structure, achieving spatial chromatic enhancement and temporal trajectory imaging. These results reveal the feasibility of AMSPA for constructing artificial vision systems.

## Graphical abstract

This study introduces retinomorphic active‐matrix synaptic phototransistor array based on metal oxide/organic semiconductor heterojunctions, incorporating in‐sensor chromatic color‐opponent processing. The dual photogates enable synaptic color‐opponent processing with a high current dynamic range exceeding 90 dB. The array enables spatial chromatic contrast imaging and dynamic trajectory capture for efficient artificial color perception.

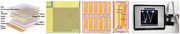

## 1 Introduction

Color recognition plays a pivotal role in extracting both spectral and spatial features across the UV to near‐infrared (NIR) spectrum, a capability crucial for the development of intelligent systems such as the Internet of Things, industrial inspection, augmented reality, and autonomous driving.^[^ ^1^, ^2^, ^3^, ^4^, ^5^, ^6^ ^]^ Current color recognition systems typically utilize complementary metal‐oxide‐semiconductor (CMOS) sensing systems with four Bayer color filters (two pixels for green, one for blue, and one for red) and an interpolation method. These systems exhibit limitations marked by the use of additional splitting prisms, information loss during color reconstruction, and a consequent reduction in pixel density.^[^ ^7^, ^8^ ^]^ Furthermore, the CMOS imaging system is restricted to only spatial frame acquisition lacking the capacity to integrate temporal data. Accurate temporal fusion imaging demands high power consumption from the high signal transmission demands between separate sensing, memory, and computing units.^[^ ^8^, ^9^, ^10^, ^11^ ^]^

Comparatively, the human retina efficiently perceives and processes temporal color images through synaptic plasticity and color‐opponent coding.^[^ ^12^, ^13^ ^]^ Opponent processing entails bidirectional responses to specific color pairs and chromatic adaptation, resulting in increased discrimination between opponent color pairs, and ultimately accelerating color perception.^[^ ^13^ ^]^ Meanwhile, the synaptic plasticity of the synapse and neuron network enables temporal signal integration and processing for low‐power, highly efficient perception. Recently, various strategies have been explored to develop retina‐inspired color vision sensors featuring spectral‐dependent bidirectional and synaptic photoresponse, providing a great potential to achieve a more efficient and dynamic color processing system than the current CMOS counterpart.^[^ ^4^, ^13^, ^14^, ^15^, ^16^, ^17^, ^18^, ^19^, ^20^, ^21^, ^22^, ^23^, ^24^ ^]^ However, due to the inherent photoelectric effect, there remain great challenges to achieving wavelength‐dependent bidirectional photoresponse in a singular device free of external electric modulation or gate bias.^[^ ^25^, ^26^ ^]^ Moreover, the dynamic range of configurable synaptic current levels (referred as current dynamic range) is quite constrained (<10 dB) of the reported bidirectionally responsive devices due to the difficult interface and material design to balance the device performance and the bidirectionally synaptic processing functionality.^[^ ^17^, ^27^, ^28^, ^29^, ^30^ ^]^

Additionally, for constructing artificial vision systems, achieving large‐area, low power consumption, and high‐density integration at the array level is critical. However, currently reported synaptic device arrays often consist of separate devices or low‐density passive arrays,^[^ ^1^, ^3^, ^31^, ^32^, ^33^ ^]^ resulting in challenges related to signal crosstalk and low spatial resolution for real‐time imaging and in‐sensor processing. In contrast, the active‐matrix (AM) configuration incorporates a thin film transistor (TFT) per unit pixel, allowing individual pixel activation through TFT control. The exceptionally low off‐current of the TFT helps mitigate signal crosstalk and reduces power consumption, addressing challenges prevalent in passive‐matrix architectures. However, integrating synaptic devices, particularly those with bidirectional response, into the active matrix for dynamic imaging and in‐sensor processing presents significant difficulties due to intricate device design, limited material options, and incompatible fabrication processes.^[^ ^16^, ^28^, ^33^, ^34^, ^35^ ^]^

Herein, we propose a facile method to achieve color‐opponent processing capability by dual‐photogate of metal oxide/organics heterojunction and demonstrate an active‐matrix synaptic phototransistor array (AMSPA) for filter‐free dynamic imaging with in‐sensor spectral processing function. The hybrid phototransistor shows bidirectional synaptic photoresponse based on specific input wavelengths, with UV‐inducing excitation and Vis/NIR‐inducing inhibition. Additionally, the synaptic phototransistor can simulate the chromatic adaptation process and show a high current dynamic range of over 90 dB, greatly addressing the low current dynamic range issue of typical bidirectional synaptic devices.^[^ ^17^, ^27^, ^28^, ^29^, ^30^ ^]^ Furthermore, through the monolithic integration of these synaptic phototransistors into an active pixel, we have successfully demonstrated their utility in enhanced spatial color contrast and dynamic trajectory imaging. This synaptic phototransistor array shows promise for constructing a compact, low‐power, and highly efficient artificial vision system.

## 2 Results

### 2.1 Organic/Inorganic Hybrid Phototransistor for Bidirectionally Synaptic Response

**Figure 1:**
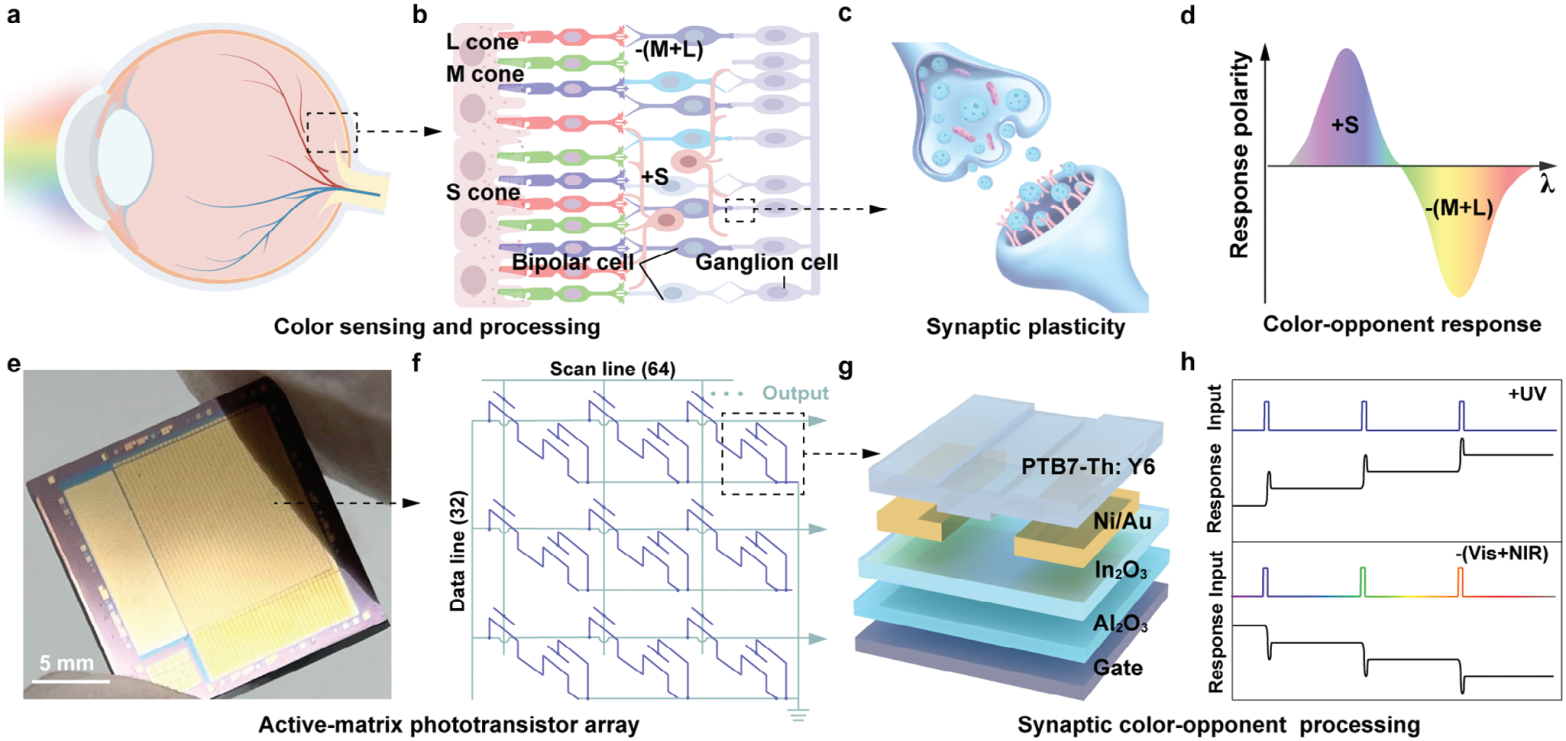
Retinal and artificial retinal structure. a) Schematic illustration of part of the human visual system. b) Illustration of the retina capable of photo‐sensing and antagonistic coding processing. c) The synaptic plasticity functionality in the synapse and neuron network. d) The retina outputs blue‐yellow opponent color pairs with the bidirectional response. e) Optical image of the AMSPA. f) The schematic representation of the AMSPA's equivalent circuit. g) The illustration of hybrid phototransistor structure configuration. h) The hybrid phototransistor processes the UV–vis/NIR opponent color pairs by bidirectionally synaptic plasticity.

The human capacity to discern a broad spectrum of colors depends on color‐sensitive receptors known as cones within the retinal tissue. These cones can be categorized into three distinct types (S cone, M cone, and L cone), each attuned to specific regions of the color spectrum, namely blue, green, or red (**Figure** **1**a,b). Despite this only three cone types, the perceptual diversity of colors is achieved through the comparative analysis of the responses generated by three cone types.^[^ ^36^, ^37^ ^]^ For example, the responses of blue‐sensitive cones (S cones) are systematically compared with those induced by green/red‐sensitive cones (M and L cones) by the synaptic plasticity functionality of the bipolar cells, thus giving rise to the perception of colors along the blue–yellow continuum (Figure 1c). The neural elements within the retinal tissue responsible for executing such comparative operations are referred to as color opponent ganglion cells. These ganglion cells can be subcategorized based on their response characteristics like blue and yellow light (the sum of green and red light) stimuli. For example, they are denoted as either blue‐ON/yellow‐OFF (+S/‐(M+L)), signifying their excitation by blue light and inhibition by yellow light (Figure 1d).

Figure 1e shows the image of the AMSPA and Figure 1f illustrates the equivalent circuit of the array with one TFT and one phototransistor (1‐T‐1‐PT) in each pixel. The phototransistor is based on In_2_O_3_/organic bulk heterojunction (BHJ) (Figure 1g). The high‐resolution cross‐section and energy‐dispersive spectroscopy (EDS) mapping of hybrid heterojunction are illustrated in Figure S1 (Supporting Information). In this setup, In_2_O_3_ primarily responds to UV stimulation, and the persistent photoconduction characteristics of In_2_O_3_ facilitate positive synaptic modulation when exposed to UV light. The BHJ, on the other hand, serves as the photoreceptor for the visible‐to‐NIR (Vis–NIR) spectrum. The minor fraction of the acceptor material within the BHJ functions acts as a photogate to decrease the conductivity of In_2_O_3_, resulting in inhibitory and synaptic responses under Vis–NIR illumination. The In_2_O_3_ and BHJ components function as UV and Vis–NIR photoreceptors, similar to the cone cells in biological systems, and the dual‐photogate including interfacial electron trapping and ionized oxygen vacancies operate analogously to bipolar and ganglion cells, thereby enabling bidirectional synaptic encoding for opponent color pairs (Figure 1h).

The incorporation of organic BHJ materials as light absorbers combing the high‐mobility channel materials has been widely investigated for high‐sensitivity heterojunction photo‐sensors.^[^ ^38^, ^39^ ^]^ Efficient exciton dissociation and carrier transport in these systems depend on an optimal band alignment and donor‐to‐acceptor (D/A) ratio in the BHJ composition.^[^ ^40^ ^]^ Herein, we substantially enhance the D/A ratio of the BHJ while integrating the In_2_O_3_ channel, thereby modifying the sensor's response to a wavelength‐dependent synaptic response. The high D/A ratio greatly increases the number of electron trapping sites within the morphologically isolated acceptor domains, facilitating effective negative photogating.^[^ ^41^, ^42^, ^43^ ^]^ Subsequently, these entrapped electrons serve as a negative photo‐gate, yielding a discernible reduction in the conductivity of the In_2_O_3_ channel, and facilitating inhibitory synaptic responses.^[^ ^17^, ^44^ ^]^ Combining the low activation energy of oxygen vacancy ionization in the In_2_O_3_ film, the excitation response under UV can be achieved, enabling the realization of synaptic color‐opponent processing.^[^ ^45^, ^46^, ^47^ ^]^

First, we ascertained the necessity of the In_2_O_3_/BHJ heterojunction for the color‐opponent synaptic functionality. In Figure S2 (Supporting Information), we presented the photoresponse characteristics of organic phototransistors with channels of Y6, PTB7‐Th, sequentially deposited PTB7‐Th/Y6,^[^ ^48^ ^]^ high D/A ratio (10:1) BHJ, and low D/A ratio (1:1.5) BHJ. Both the transient photoresponse and post‐synaptic current (EPSC) exhibited values less than 1 nA, indicative of limited photoresponse efficacy. Figure S3 (Supporting Information) depicts the photoresponse characteristics of hybrid phototransistors with solution‐processed In_2_O_3_ channels. Under UV illumination, these phototransistors exhibited excitatory long‐term plasticity attributed to persistent photoconductivity. In contrast, under green and NIR light, the In_2_O_3_/PTB7‐Th and In_2_O_3_/BHJ (high D/A ratio) configurations displayed inhibitory synaptic plasticity. It is noteworthy that heterojunction phototransistors exhibited an overall higher photocurrent and memorized current than their organic counterparts. The In_2_O_3_/BHJ phototransistor, particularly that with a high D/A ratio, demonstrated the most pronounced inhibitory post‐synaptic current (IPSC) under green and NIR light, albeit at the expense of the lower UV synaptic photocurrent (Figure S4, Supporting Information).

### 2.2 Modulation from Sensing to Bidirectionally Synaptic Sensing

**Figure 2:**
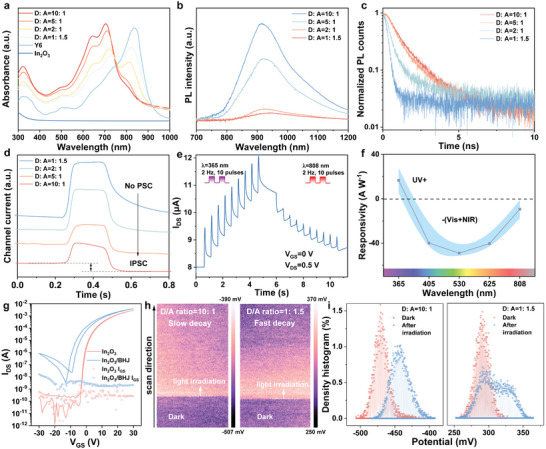
The properties of hybrid phototransistors with various heterojunction compositions. a) The absorbance of BHJ and In_2_O_3_ thin films. b) PL spectra of organic films with varying D/A ratios. c) PL decay profiles in organic films with diverse D/A ratios. d) Channel current variations in In_2_O_3_/BHJ phototransistors with different D/A ratios under a series of NIR pulses. e) The typical excitation and inhibition behavior of the hybrid phototransistor (D:A = 10:1) under UV and NIR. f) The responsivity of hybrid phototransistors to various wavelengths (D:A = 10:1). g) The transfer curves of the In_2_O_3_ transistor and In_2_O_3_/BHJ (D:A = 10:1) hybrid transistor. h) Mapping CPD in high (left) and low D/A ratio (right) In_2_O_3_/BHJ hybrid channels. During the scanning process, it undergoes a sequence that starts with dark conditions, followed by the illumination of NIR light, and then the removal of the light source. i) Particle analysis of the dark regions and regions following a 180 s illumination period of the KPFM results for two distinct cases:D/A ratio of 10:1 (left) and D/A ratio of 1:1.5 (right).

Subsequently, we conducted further investigations into the impact of varying D/A ratios on the hybrid phototransistor to observe the transition process from sensing performance to synaptic performance. **Figure** **2**a illustrates the absorbance spectra of PTB7‐Th:Y6 blend films with varying D/A ratios. The absorbance spectra of these blend films manifest three distinctive absorbance peaks. As the proportion of the donor component increases, there is a discernible enhancement in the intensity of the shoulder peaks observed at 640 and 700 nm, while the intensity of the shoulder peak at 835 nm gradually diminishes. The polymer donor significantly contributes to absorbance within the visible light spectrum, whereas Y6 plays a more prominent role in the NIR range.^[^ ^48^, ^49^ ^]^ In contrast, the wide bandgap material In_2_O_3_ demonstrates absorbance in the UV range while maintaining transparency for wavelengths longer than 400 nm. Figure S5 (Supporting Information) illustrates the surface morphologies of the BHJ films and In_2_O_3_ films. The BHJ blend films, characterized by varying D/A ratios, consistently displayed smooth surfaces with root‐mean‐square roughness (R_q_) values in the vicinity of ≈0.7 nm. This observation signifies a high degree of miscibility between Y6 and PTB7‐Th. The surface of solution‐processed In_2_O_3_ also exhibited a smooth topography, contributing to the reduction of interfacial scattering. In contrast, the pristine Y6 film exhibited a higher *R*_q_ value of 1.64 nm, indicative of greater crystallinity.

Steady‐state photoluminescence (PL) spectra were analyzed to investigate the exciton dissociation and charge transfer characteristics within the BHJ material, across a range of D/A blend ratios (Figure 2b). When the D/A weight ratio is 2:1 or 1:1.5, the PL peak exhibits substantial quenching in comparison to the BHJ film with a higher D/A ratio. This quenching signifies the efficient transfer of electrons from the PTB7‐Th donor to the Y6 acceptor, indicating the efficacy of charge transfer processes within the BHJ. Comparatively, the high D/A ratio (5:1, 10:1) BHJ films have a high PL peak indicating the inferior charge transfer process. In Figure 2c, the time‐resolved photoluminescence (TRPL) measurements were conducted on BHJ with various D/A ratios (excitation wavelength:510 nm, pulse time:10 ns). In Figure S6 (Supporting Information), the decay lifetimes were extracted through data fitting using a biexponential decay model, defined as follows:

(1) $$y=A_{1}\;\exp\left(-t/\tau_{1}\right)+A_{2}\;\exp\left(-t/\tau_{2}\right)$$

where *A*_1_ and *A*_2_ represent the relative amplitudes, while τ₁ and τ₂ denote the lifetimes associated with the rapid and gradual recombination processes, respectively. The obtained fitting parameters (A_1_ and A_2_) and decay lifetimes (τ_1_ and τ_2_) are summarized in Table S1 (Supporting Information). The average lifetime (τ_a_) was subsequently calculated based on the extracted data using the following formula:^[^ ^50^ ^]^

(2) $$\tau_{a}=\frac{\sum_{i}A_{i}\tau_{i}^{2}}{\sum_{i}A_{i}\tau_{i}}$$

The analysis revealed that the values of *τ*_1_ and *τ*_a_ for the BHJ exhibited a progressive increase as the donor ratio was elevated. Notably, when the D/A ratio reached 10:1, the quick and average decay lifetimes were measured at 0.91 and 2.11 ns, respectively, which significantly exceeded those observed in the 1:1.5 D/A ratio film (*τ*_1_ = 0.40 ns, *τ*_a_ = 0.19 ns). The extension in lifetime can be attributed to the creation of more incoherent acceptor domains, facilitating electron trapping and inhibiting charge transfer.

The photoresponse characteristics of In_2_O_3_/BHJ hybrid phototransistors with varying D/A ratios are shown in Figure 2d. Notably, following each NIR pulse irradiation, the baseline current exhibited a persistent long‐term depression (LTD), an effect that became more pronounced with increasing D/A ratios, where the sensor‐type property gradually changed to the synaptic property. A similar trend is observed in the photoresponse of the hybrid phototransistors under red, green, and blue light (Figure S7, Supporting Information). In Figure 2e, the temporal variation of the channel current is depicted under continuous UV and NIR light pulses. The observed current behavior comprises two distinct processes:initial temporal increase followed by a subsequent baseline increase (UV) or decrease (NIR). Figure 2f shows the responsivity (R) calculated from the post‐synaptic current (PSC) after 150 pulses under various wavelength irradiations (pulse width:100 ms, power density:0.2 mW cm^−2^, 15 s irradiation time in total). Notably, the UV response exhibits a positive polarity with a responsivity of 16.6 A W^−1^. Conversely, negative polarities are observed under visible and NIR light, with responsivity values of −39.0, −49.0, −40.4, and −9.4 A W^−1^ for blue, green, red, and NIR light, respectively. Figure 2g shows the bare In_2_O_3_ and In_2_O_3_/BHJ hybrid phototransistor with D:A = 10:1, the hybrid device displays a negatively shifted threshold voltage (V_th_), elevated off‐current, and increased subthreshold swing (SS). These characteristics are ascribed to the traps induced by the organic BHJ material and the organic current pathway.

Furthermore, we conducted Kelvin probe force microscopy (KPFM) testing to assess the surface potential changes of In_2_O_3_/BHJ channel surface following light stimuli. The local contact potential difference (CPD) mapping of BHJ films with high and low D/A ratios on In_2_O_3_ is presented in Figure 2h. First, the NIR pulse was applied, and the surface potential increased. Subsequently, the light was removed, and the surface scanning continued to observe the decay performance without any external electrical bias. Statistical histograms were also extracted for both the dark conditions and the regions scanned after light source removal (Figure 2i). The high D/A ratio hybrid films exhibited a rise in surface potential from an initial state of ≈−472 to ≈−443 mV. The histograms of both regions still displayed distinct peaks after 180 s of decay, indicative of a long carrier lifetime. In contrast, the low D/A ratio hybrid films demonstrated a higher increase, transitioning from ≈290 to ≈328 mV, while concurrently exhibiting a faster decay as discerned from the portion of the histogram distribution returning to the initial pristine potential.

### 2.3 Color‐Opponent and Chromatic Adaptation

**Figure 3:**
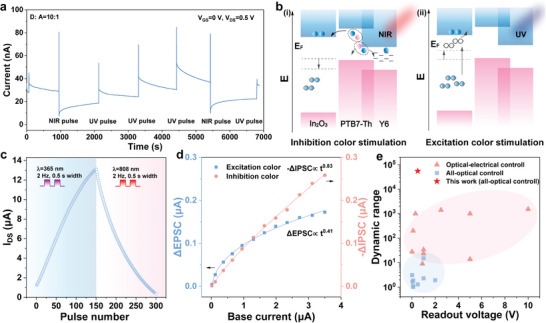
The properties of hybrid phototransistors with various heterojunction compositions. a) The channel current under a series of NIR and UV pulses. b) The schematic diagram of the operational mechanism of the hybrid phototransistor under inhibition color stimulation (i) and excitation color stimulation (ii). c) The channel current variation under succussive UV and NIR pulses. d) The photocurrent variation to the NIR and UV pulse at different base channel currents with alternated UV/NIR stimulation. e) The comparison of the readout voltage and current dynamic range with recently reported synaptic optoelectronic devices. The extracted current dynamic range covers both excitatory and inhibitory processes, providing the overall adjustable range.

**Figure** **3**a shows a long‐time test under excitation or inhibition color irradiation to elucidate the bidirectional synaptic property of the high D/A ratio hybrid phototransistor. The proposed underlying mechanism is elucidated in Figure 3b. Upon inhibition of color irradiation (visible or NIR light), the BHJ serves as the primary absorber, with PTB7‐Th for visible light and Y6 for NIR light. When NIR light is applied, excitons are generated, which subsequently dissociate into free electrons and holes (Figure 3bi). These holes can be directly collected by the electrodes, while the released electrons migrate energetically to trapping sites within the isolated Y6 domains. Here, they function as negative photogates, reducing the conductivity of the In_2_O_3_ film and leading to IPSC. Simultaneously, a fraction of electrons flow into the In_2_O_3_, contributing to the transient photoresponse. The processes of charge transfer and charge trapping are antagonistic and reliant on the density of occupied trapping sites within the BHJ. This opposing process endows the hybrid phototransistor with adaptation behavior to the inhibition wavelength, as illustrated in Figure S8 (Supporting Information). As the trapping sites gradually become occupied, the long‐term IPSC decreases with faster decay, while the transient current increases. Similarly, a smaller number of available trapping sites (low D/A ratio) leads to decreased IPSC variation and increased transient photocurrent, as demonstrated in Figures S4 and S7 (Supporting Information). When exposed to UV light, the In_2_O_3_ film undergoes oxygen vacancy ionization, releasing free electrons and consequently increasing conductivity (Figure 3bii).

Figure 3c demonstrates the dynamic control of conductivity through the application of opponent color pairs, illustrating the all‐optical modulation capabilities of the phototransistor. Nonlinear behavior is observed in both the UV excitation and NIR inhibition processes. This phenomenon can be explained by electron accumulation resulting from prolonged UV irradiation, which renders the ionization of oxygen vacancies ineffective. During the NIR inhibition process, trapping sites become gradually occupied, decelerating the trapping mechanism. This represents the adaptation process to both excitation and inhibition stimuli. Subsequently, we investigated the chromatic adaptation capabilities of our hybrid phototransistors, focusing on the mutual influence between responses to opponent color pairs. This investigation involved the alternate application of UV and NIR light to observe their combined effects (Figure S9, Supporting Information). At various current levels, identical UV and NIR light pulses were applied to examine the PSC. The inhibitory and excitatory currents under the same light power across different baseline currents are presented in Figure 3d. Differently from the observation to the single spectral stimulation, the PSC change did not reach saturation and decreased but gradually increased (ΔEPSC ∝ t^0.41^, −ΔIPSC ∝ t^0.83^). This indicates that irradiation with one color can enhance the response to the opponent color, which is a key function of chromatic adaptation in the human visual system.

Prolonged UV exposure leads to the release and accumulation of electrons due to the ionization of oxygen vacancies. This electron accumulation, analogous to the process of color fatigue, increases the interfacial barrier and facilitates the trapping effect. The gradual decline in transient photocurrent also signifies the gradual dominance of the trapping effect over the charge transfer mechanism as illustrated in Figure S10 (Supporting Information). Similarly, after NIR irradiation, the accumulation of trapped electrons in organic materials acts as a negative ‘photogate,’ enhancing the EPSC upon subsequent UV light irradiation. This dynamic interplay mirrors the chemical reactions in the human retina during chromatic adaptation. Thus, extended exposure to UV or NIR light enhances the photoresponse to its opponent colors (NIR or UV), demonstrating the device's ability to adapt and respond to various color stimuli. To exclude the gas/water‐induced negative photoresponse, we also used the parylene C to passivate the hybrid device, and a similar negative response was observed (Figure S11, Supporting Information).

To further assess the synaptic performance, we examined the influence of the input light frequency (Figure S12, Supporting Information). High‐frequency stimuli, on the other hand, resulted in strong excitation leading to visual fatigue and decreased inhibitory current efficiency. The light power dependence has also been investigated as shown in Figure S13 (Supporting Information). Short‐term plasticity can also be achieved when the trapping sites are occupied at high *V*_GS_ (Figure S14, Supporting Information). Chromatic adaptation can enhance color contrast while concurrently reducing the linearity of synaptic current changes. In this context, we undertook adjustments to pulse width to achieve optimal linearity in the modulation of channel conductivity. We modified the NIR pulse width as the baseline current decreases, favorable linearity can still be achieved even at elevated current levels, as depicted in Figure S15 (Supporting Information). The assessment of the current dynamic range of the synaptic devices plays a critical role in the evaluation of the color‐opponent effect for achieving high‐contrast imaging.^[^ ^38^, ^51^ ^]^ In Figure S16 (Supporting Information), the current dynamic range of the UV excitatory process is presented across various baseline current levels ranging from 0.1 nA to 2 µA. The response to UV light gradually reached saturation at ≈3 µA, and the current dynamic range exceeded 10^3^ when the baseline current was less than 10 nA. For NIR inhibitory stimuli at baseline current from 0.1 nA to 2 µA, as the baseline current increased, the color adaptation effect induced a more pronounced inhibitory response. The current dynamic range achieved almost 10^3^ when the baseline current exceeded 10 nA. The extracted current dynamic range values are displayed in Figure S17 (Supporting Information), including the collective dynamic range resulting from both excitatory and inhibitory processes at different baseline levels. Noteworthy, the maximum current dynamic range achieved 5.5×10^4^ (94.4 dB) at a 10 nA baseline, a better performance compared to the most of presently reported current dynamic ranges of optoelectronic synaptic devices as illustrated in Figure 3e. Because the dynamic response of the hybrid device is determined by the trapping and ionization processes of the heterojunction, the same dynamic range can be achieved under various power densities. For lower power densities, this can be achieved by increasing the irradiation time, as illustrated in Figure S18 (Supporting Information).

Next, we consider the impact of drain and gate voltages on the performance of the phototransistor. As depicted in Figure S19 (Supporting Information), the UV response is observed following one‐second gate and drain voltage pulses of ±20 V. Notably, a transition from an excitatory to an inhibitory UV response is observed after the −20 V V_GS_ pulse. This phenomenon can be explained as the negative gate pulse inducing the release of trapped electrons within the BHJ, thereby resetting the device to its pristine state. Therefore, a portion of the incident UV light absorbed by the BHJ leads to a predominant trapping effect. After the application of the drain voltage pulse, the photoresponse experiences minimal alteration. In the case of NIR response, a similar facilitated IPSC is observed following the −20 V gate pulse, as illustrated in Figure S20 (Supporting Information). Additionally, after the application of a high drain voltage of 20 V, the inhibitory synaptic current is also facilitated. This can be explained that, during the high drain voltage pulse, electrons become trapped at the interface between the BHJ and the drain electrode. Following the removal of the bias, these trapped electrons are gradually released, resulting in facilitated electron trapping within the acceptor domain. Comparing the nearly unaltered photoresponse under UV illumination following a high drain voltage pulse, it also becomes evident that the UV response and NIR‐induced trapping mechanisms are distinct phenomena occurring within individual film domains, and the color‐opponent functionality can be flexibly modulated by the voltage pulse. The performance of the reference In_2_O_3_ TFT under varying V_DS_ and V_GS_ pulse is illustrated in Figure S21 (Supporting Information).

To further examine the influence of electric fields on photoresponse, we employed a focused light source (area of ≈3 µm^2^) to selectively irradiate different regions of a long‐channel device (100 µm), as depicted in Figure S22 (Supporting Information). Notably when the light spot (530 nm) was positioned at the center of the channel as opposed to the electrode region, we observed higher transient photocurrents and faster decay. This phenomenon can be attributed to the enhanced carrier collection facilitated by a higher drain voltage. Conversely, in the middle region where the electric field is relatively lower, the trapping effect becomes more prominent, leading to limited carrier collection. Given the drain voltage effect on the device performance, we test the bidirectional photoresponse under high‐*V*_DS_ conditions (10 V) in Figure S23 (Supporting Information). Under high drain voltage conditions, the elevated drain voltage improved both IPSC and EPSC, while also delaying UV‐induced current saturation due to enhanced carrier collection efficiency. However, as the base current was also lifted at a higher drain voltage, the dynamic range remained unimproved. In Figure S24 (Supporting Information), we confirmed the good cyclic operational stability over ten cycles, each involving 20 pulses of UV and NIR light pulses.

### 2.4 Active‐Matrix Synaptic Phototransistor Array

**Figure 4:**
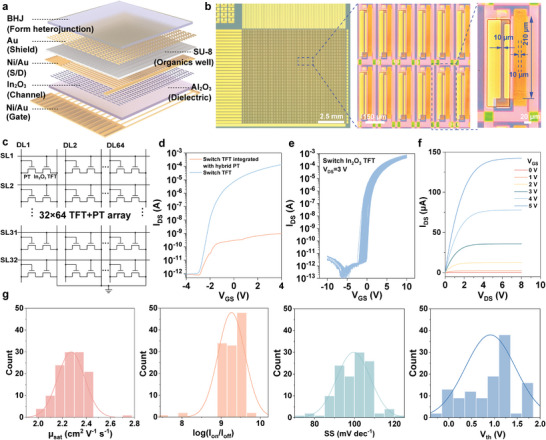
Active‐matrix synaptic phototransistor array. a) Illustrative configuration of the 32×64 AMSPA. b) Optical images of the entire array, an enlarged segment of the array, and a single pixel within the array. c) The schematic representation of the hybrid phototransistor array's equivalent circuit. Each pixel consists of a switching TFT and a hybrid phototransistor. d) The transfer curve of the switching TFT and its characteristics following integration with the hybrid phototransistor. e) 120 transfer curves of switch In_2_O_3_ TFTs in the AMSPA. f) The typical output curve of the switch In_2_O_3_ TFT. g) Statistical parameters including µ_s__at_, I_on_/I_off_ ratios, SS, and V_th_ for a set of 120 In_2_O_3_ switch TFTs.

Subsequently, we integrated our hybrid synaptic phototransistors into an active matrix, as depicted in **Figure** **4**a. The fabrication process is outlined in Figure S25 (Supporting Information), and a regional array of optical images corresponding to each step of the fabrication process is provided in Figure S26 (Supporting Information). An enlarged AMSPA image is presented in Figure 4b. The W/L (210/10 µm) of the switch In_2_O_3_ TFT is higher than that of the load hybrid (In_2_O_3_/BHJ) phototransistor (190/10 µm) for effective readout control. This AMSPA has 32×64 (2048) pixels within a 1×1 cm^2^ area, and the calculated pixel per inch (PPI) is 128, and the detailed comparison with the optoelectronic device array is shown in Table S2 (Supporting Information).^[^ ^2^, ^3^, ^4^, ^16^, ^28^, ^32^, ^33^, ^35^, ^36^, ^38^, ^39^, ^52^, ^53^, ^54^ ^]^ Each pixel comprises one In_2_O_3_ switch TFT and one hybrid phototransistor (1‐T‐1‐PT), as depicted in the equivalent circuit diagram in Figure 4c. Here, the scan line applies the voltage (*V*_SL_) onto the switch TFT gate, while the data line applies the readout voltage (*V*_DL_). 100 nm Au is evaporated to shield the channel of the switch TFT to avoid unnecessary photocurrent during the sensing process (Figure S27, Supporting Information). ≈2 V V_th_ shift has been observed under 20 mW cm^−2^ UV irradiation, indicating good shield performance.

The hybrid phototransistor is configured in diode mode, benefiting from the bidirectional photoresponse at zero gate voltage, to reduce the leads and reduce the write/read operation complexity. The gate electrode of the hybrid phototransistor is normally connected to the ground with the source electrode, and the reset operation by the negative gate voltage can also be applied (Figure S20, Supporting Information). Figure 4d displays the typical transfer curves of In_2_O_3_ TFTs before and after integration with an In_2_O_3_/BHJ hybrid phototransistor. The channel current is constrained due to the high resistance of the hybrid phototransistor in its state. The transfer curves of 120 switch TFTs within the AMSPA are presented in Figure 4e, and the typical output curve is shown in Figure 4f. The corresponding extracted parameters including V_th_, current on/off ratio, saturation field‐effect mobility (µ_s__at_), and SS are demonstrated in Figure 4g, revealing uniform device performance. The statistics are concluded in Table S3 (Supporting Information). When the switch TFT is in the off state (gate voltage < 1 V), the synaptic current of the hybrid phototransistor cannot be read out, and the pixel current can be as low as 1 pA, significantly avoiding pixel crosstalk. In contrast, when the switch TFT is turned on, upon the read voltage (*V*_DL_) is applied, the postsynaptic current becomes accessible for readout.

**Figure 5:**
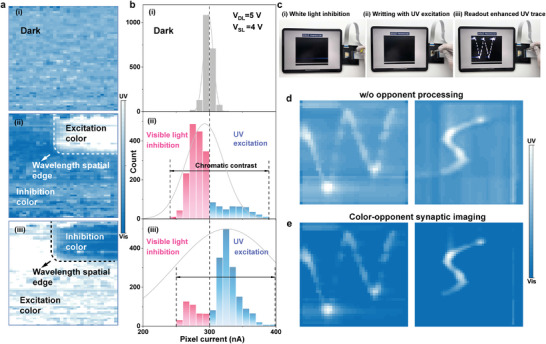
In‐sensor spectral processing. a,b) The obtained current mapping (a) and corresponding histograms (b) of the synaptic transistor array under dark, global inhibition and regional excitation, global excitation, and regional inhibition. The inhibition light source is white light while the excitation light source is the UV light. c) The measurement of the synaptic phototransistor array capturing enhanced UV light moving trace. d) The trace obtained without wavelength‐dependent response function. e) The normalized readout current mapping of enhanced UV movement trajectory of letter pattern “W” and “S”.

Finally, we employed the color‐opponent AMSPA for spatial chromatic enhancement and temporal writing trace imaging. The AMSPA was connected to the interface PCB board via wire bonding and then linked to the source and matrix switch for real‐time imaging, as detailed in Figure S28 (Supporting Information). Initially, dark current mapping (2048 pixels) was conducted, with readout at *V*_DL_ = 5 V and *V*_SL_ = 4 V (**Figure** **5**a(i)). The mean dark current value was measured to be ≈300 nA (Figure 5b(i)). Subsequently, global white light was applied to irradiate the array, with a rectangular area exposed to UV light. The resultant image in Figure 5a(ii) clearly distinguishes chromatic spatial edges through opponent color‐processing. The corresponding histogram indicates a negative shift in the inhibition color‐irradiated area compared to the dark value. Conversely, the partially irradiated excited area shows a positive shift, exhibiting a higher mean value. In another test, the AMSPA was globally irradiated with UV light while the rectangular area was exposed to white light. In this scenario, clear wavelength spatial edges were also observed (Figure 5a(iii)). The corresponding histogram (Figure 5b(iii)) also displays a double‐directional shift, akin to the previous case. Henceforth, pixels with currents below the threshold of 300 nA can be discerned as indicative of visible/NIR light input, whereas those surpassing this threshold can be distinguished as UV light input, obviating the necessity for wavelength‐specific filters. Furthermore, in contrast to conventional color image sensors with only a positive response, the bidirectional response can enhance the spatial chromatic contrast, denoting the amplitude disparities of diverse color channels (Figure S29, Supporting Information).

Furthermore, we integrate color‐opponent processing with synaptic properties to demonstrate the trajectory record function. As depicted in Figure 5c, we initially used global white light to irradiate the AMSPA to enhance the following UV information by chromatic adaptation (i), and then the UV laser was applied to “write” a letter over the AMSPA (ii). Following the writing process, we sequentially scan the pixels of the array to read out the memorized trace. The written letter “W” can be read out (iii) (Video S1, Supporting Information). Without opponent processing, the simultaneous input of dual‐band light could interfere with the other wavelength information mutually, leading to significant noise generation by the opponent color, as shown in Figure 5d. In comparison, using AMSPA, the inhibition color can be greatly suppressed, and the spatial information carried by the excitation color can be accurately enhanced and recorded through chromatic adaptation. This allows the UV trace to be enhanced and read out after the moving process and distinguished as written “W” and “S” (Figure 5e). Under low irradiation condition, the pixel current variation can degrade imaging quality, while the imaging quality can be improved by extending irradiation time (Figure S30, Supporting Information).

## 3 Conclusion

We propose an active‐matrix synaptic phototransistor array (32×64) with a 1‐T‐1‐PT structure based on In_2_O_3_/PTB7‐Th:Y6 heterojunction. The BHJ composition modulation enables dual‐photogate for in‐sensor color opponent processing and chromatic adaptation, resulting in a high current dynamic range exceeding 90 dB for high‐contrast color perception. The AMSPA demonstrates enhanced spatial chromatic contrast and temporal trace imaging via synaptic color‐opponent processing and chromatic adaptation. This approach is feasible for constructing artificial vision systems for efficient in‐sensor spectral processing.

## 4 Experimental Section

### Device Array Fabrication

A 2‐inch polished silicon wafer coated with SiO_2_ was utilized as the substrate material. The substrate underwent a comprehensive cleaning process involving the following steps:ultrasonication in Standard Clean 1 (SC‐1, NH_4_OH:H_2_O_2_:H_2_O, v:v = 1:2:7), rinsing with deionized water, ultrasonication in Standard Clean 2 (SC‐2, HCl:H_2_O_2_:H_2_O = 1:1:6), and another deionized water rinse. Each of these cleaning steps was conducted for a duration of 30 min at a temperature of 70 °C. Subsequently, a 5/30 nm Ni/Au bilayer was deposited using e‐beam evaporation to serve as the bottom gate material, and the pattern was defined through a lift‐off process. Following this, a 30 nm thick Al_2_O_3_ layer was deposited using plasma‐enhanced atomic layer deposition (PEALD) employing trimethylaluminium (TMA) at an operating temperature of 250 °C.

The preparation of the In_2_O_3_ precursor solution involved dissolving indium nitrate in 2‐methoxyethanol (2‐ME) to achieve a concentration of 0.1 m. Additionally, acetylacetone (AcAc) and ammonium hydroxide (NH_3_·H_2_O) were added at an equimolar concentration to act as fuel, facilitating the exothermic combustion reaction. Following a 10‐min O_2_ plasma treatment, the In_2_O_3_ precursor solution was spin‐coated at 3000 rpm for 30 s (relative humidity<50%), pre‐baked at 200 °C for 10 min, subjected to photolithography patterning, and etched using HCl:H_2_O (1:15, v:v) for 3 s to form the channel patterns for two TFTs within each pixel. The In_2_O_3_ patterns were subsequently annealed at 300 °C for 1 h.

Subsequently, the Al_2_O_3_ layer was etched using H_3_PO_4_:H_2_O (v:v = 1:1) for 10 min at 40 °C to reveal the extended gate electrodes and the local gate of the phototransistor. An 8/50 nm Ni/Au bilayer was deposited using e‐beam evaporation as the source and drain (S/D) electrodes, with the pattern defined through a lift‐off process. This defined the W/L ratio as 190/10 µm for the phototransistor and 210/10 µm for the switch TFT. Subsequently, an SU‐8 (2000.5) film was deposited and patterned using photolithography techniques to enable the subsequent deposition of organic absorbers. An 8/50 nm Ni/Au layer was then e‐beam evaporated to function as the UV shield for the switch TFT. Lastly, a series of PTB7‐Th:Y6 solutions were prepared by dissolving a total of 9 mg mL^−1^ of the material (in ratios of PTB7:Y6 = 10:1, 5:1, 2:1, and 1:1.5) in trichloromethane and applied through dynamic spin‐coating followed by 5 min annealing at 110 °C.

### Film and Device Fabrication and Characterization

The roughness values of the organic films and KPFM were determined through atomic force microscopy (AFM) using a Bruker Dimension ICON instrument. The optical transmittance of the films was assessed using a UV–vis spectrophotometer (UV‐2700, Shimadzu). The Al_2_O_3_ film was deposited via atomic layer deposition (ALD) utilizing the SI ALD system from SENTECH Instruments GmbH. PL was characterized using a photoluminescence spectrometer (FLS1000, Edinburgh Instruments). High‐resolution cross‐sections were acquired with a transmission electron microscope (Talos L120C, Thermo Fisher). The wire bonding was conducted with the wire bonder of 7KE, WEST BOND. Electrical characteristics were evaluated employing a semiconductor parameter analyzer (Keithley 4200A‐SCS and/or Keysight B2912A). The devices were illuminated with monochromatic light from different LEDs or lasers (365, 405, 530, 625, and 808 nm, corresponding to UV, blue, green, red, and NIR light, respectively), which were driven by a signal generator (Tektronix AFG3152C). The light intensity was calibrated using a standard silicon photodiode (Thorlabs S120VC). CPD is created between a tip and the device surface, which can be used to quantify the work function via the following equation:

(3) $$V_{CPD}=\frac{W_{tip}-W_{sample}}{e^{-}}$$

where *W*_tip_ and *W*_sample_ are the work functions of the tip and sample surface, respectively. The TFT parameters are extracted as follows:

(4) $$\mu_{\mathrm{sat}}=\frac{2L}{WC_{ax}}{\left(\frac{\partial \sqrt{I_{DS}}}{\partial V_{GS}}\right)}^{2}$$

(5) $$SS=\frac{\partial V_{GS}}{\partial \left(\mathrm{lg}\;I_{DS}\right)}$$

### Active‐Matrix Phototransistor Array Measurement

An NI PXIe‐1073 PXI chassis, incorporating the NI PXIe‐2531 PXI matrix switch module and the NI PXIe‐4138 PXI source measure unit, was employed for the acquisition of array data. Control, processing, and readout procedures were realized using LabVIEW. The source, drain, and gate of the phototransistor array were connected to a customized PCB via wire bonding (97 leads in total). The interface board was directly linked to the matrix switch and source using cables. The resulting images were further processed through thresholding and smoothing filtering techniques.

## Conflict of Interest

The authors declare no conflict of interest.

## Supporting information

Supporting Information

Supplemental Video 1

## Data Availability

The data that support the findings of this study are available from the corresponding author upon reasonable request.
