# Supplementary material for: An Active‐Matrix Synaptic Phototransistor Array for In‐Sensor Spectral Processing
Source: Adv Sci (Weinh). 2024 Aug 21;11(39):2406401. doi: 10.1002/advs.202406401 (PMC11497057; doi:10.1002/advs.202406401)
Supplement: Supplementary file 1 — Supporting Information [file ADVS-11-2406401-s002.docx]

Supporting Information

**Active-Matrix Synaptic Phototransistor Array for In-Sensor Spectral Processing**

*Dingwei Li, Yitong Chen, Huihui Ren, Yingjie Tang, Siyu Zhang, Yan Wang, Lixiang Xing, Qi Huang, Lei Meng, Bowen Zhu**

Mr. D. Li, Ms. S. Zhang, Dr. L. Xing, Dr. Q. Huang, Prof. B. Zhu

Westlake Institute for Optoelectronics, Hangzhou 311421, China.

Mr. D. Li, Ms. Y. Chen, Ms. H. Ren, Mr. Y. Tang, Ms. S. Zhang, Ms. Y. Wang, Prof. B. Zhu

Key Laboratory of 3D Micro/Nano Fabrication and Characterization of Zhejiang Province, School of Engineering, Westlake University, Hangzhou 310024, China.

Mr. D. Li, Ms. Y. Chen, Ms. H. Ren, Mr. Y. Tang, Ms. Y. Wang

College of Information Science and Electronic Engineering, Zhejiang University, Hangzhou 310027, China.

Prof. L. Meng

Beijing National Laboratory for Molecular Sciences, CAS Key Laboratory of Organic Solids, Institute of Chemistry, Chinese Academy of Sciences, 100190, Beijing, China

Prof. B. Zhu

Institute of Advanced Technology, Westlake Institute for Advanced Study, Hangzhou 310024, China.

^*^ Corresponding author. E-mail: zhubowen@westlake.edu.cn;

**Keywords:** optoelectronic synapse, active-matrix array, bidirectional photoresponse, bulk heterojunction


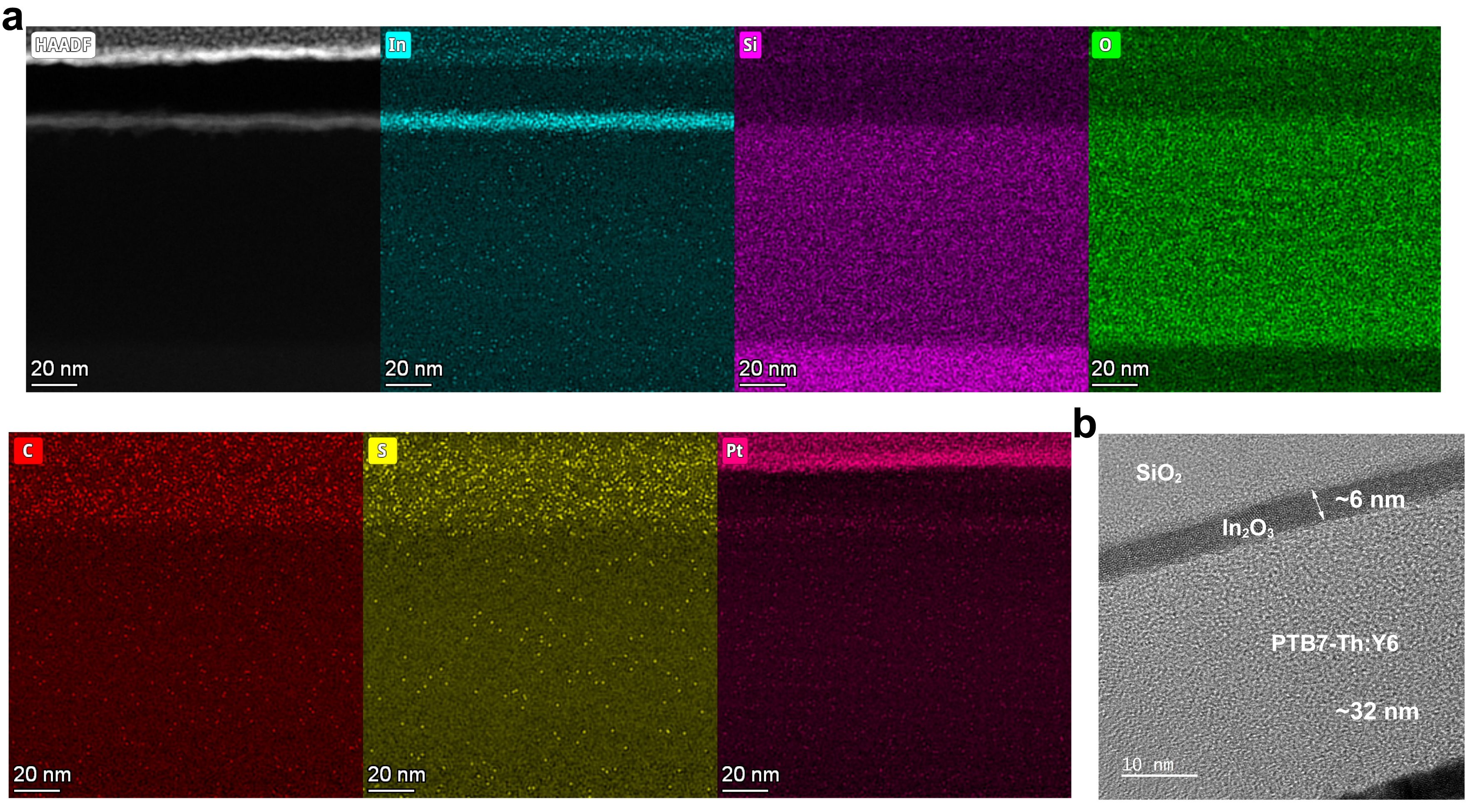
Figure S1. The high-resolution cross-section of hybrid structure. (a) High-resolution EDS mapping of the elements indium (In), silicon (Si), oxygen (O), carbon (C), sulfur (S), and platinum (Pt). (b) Cross-section TEM image of In_2_O_3_/BHJ heterojunction on SiO_2_ dielectric.


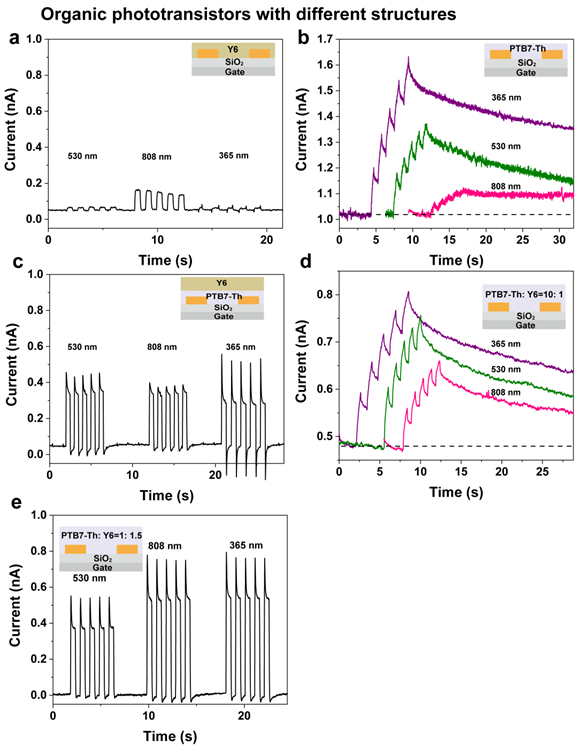


**Figure S2**. **Photoresponse of various organic phototransistors.** The photoresponse characteristics of various organic phototransistors under a sequence of 5 pulses (1 Hz, pulse width of 500 ms) of light at different wavelengths, specifically green (530 nm, 0.2 mW cm^-2^), NIR (808 nm, 0.8 mW cm^-2^), and UV (365 nm, 2 mW cm^-2^) at V_GS_=0V and V_DS_=0.5 V. (**a**) The Y6 phototransistor. (**b**) The PTB7-Th phototransistor. (**c**) The layer-by-layer deposited PTB7-Th/Y6 phototransistor. (**d**) The BHJ phototransistor with high D/A ratio (PTB7-Th: Y6=10: 1). (**e**) The BHJ phototransistor with low D/A ratio (PTB7-Th: Y6=1: 1.5).

Notably, Y6, PTB7-Th/Y6, and the low D/A ratio phototransistors exhibited characteristic photosensor behaviors under UV, green, and NIR light stimuli. In comparison, PTB7-Th and high D/A ratio phototransistors demonstrated excitatory EPSC.


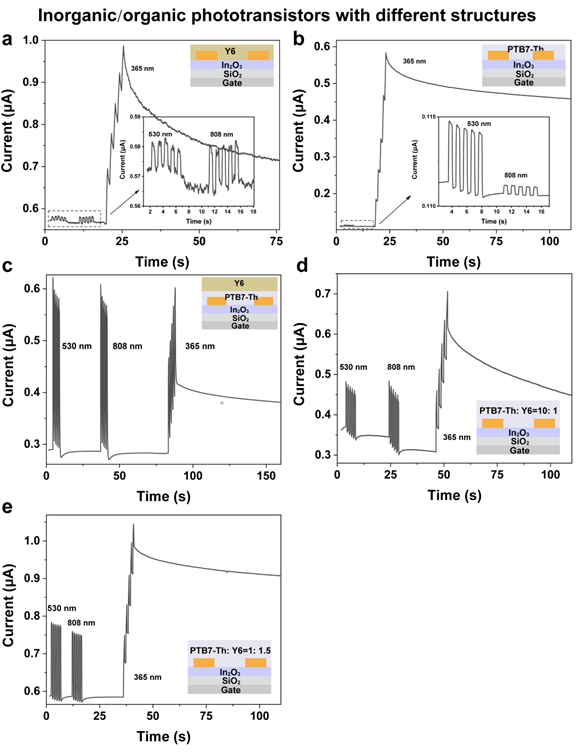
**Figure S3**. **Photoresponse of inorganic/organic phototransistors.**  The photoresponse characteristics of various In_2_O_3_/organic phototransistors subjected to a sequence of 5 pulses (1 Hz, pulse width of 500 ms) of light at different wavelengths, specifically green (530 nm, 0.2 mW cm^-2^), NIR (808 nm, 0.8 mW cm^-2^), and UV (365 nm, 2 mW cm^-2^) at V_GS_=0V and V_DS_=0.5 V. (**a**) The In_2_O_3_/Y6 phototransistor. (**b**) The In_2_O_3_/PTB7-Th phototransistor. (**c**) The In_2_O_3_ with layer-by-layer deposited PTB7-Th/Y6 hybrid phototransistor. (**d**) The In_2_O_3_/BHJ phototransistor with high D/A ratio (PTB7-Th: Y6=10: 1). (**e**) The In_2_O_3_/BHJ phototransistor with low D/A ratio (PTB7-Th: Y6=1: 1.5).


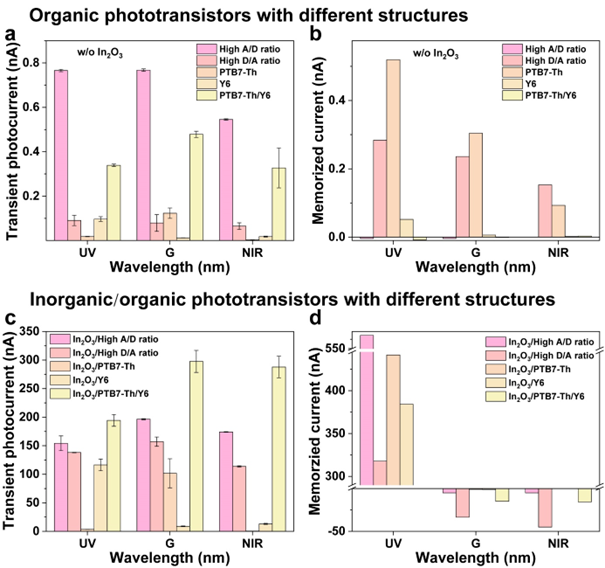
**Figure S4**. **The extracted transient and memorized photocurrent of different.** (**a**) The transient photocurrent of organic phototransistors. (**b**) The memorized photocurrent of the organic phototransistors. (**c**) The transient photocurrent of hybrid phototransistors. (**d**) The memorized photocurrent of the hybrid phototransistors.

In the absence of the In_2_O_3_ channel layer, the organic phototransistors exhibited inferior transient and memorized photocurrent. Conventional low D/A ratio (1: 1.5) BHJ organic phototransistors demonstrated superior transient photocurrent behavior, as evidenced in Figure S4a. Furthermore, PTB7-Th and high D/A ratio BHJ phototransistors displayed the highest EPSC.

Upon the incorporation of In_2_O_3_ to enhance the charge transport, a significant overall enhancement in photocurrent was observed. Nevertheless, the high D/A BHJ hybrid phototransistor displayed the highest IPSC, even though it exhibited a slight reduction in IPSC under UV illumination.

The increased EPSC of PTB7-Th and high D/A BHJ phototransistors can be attributed to the trapping of electrons in the p-type channel region. With the inclusion of the n-type In_2_O_3_ channel, these trapped electrons act as negative photo-gates, thereby reducing channel conductivity and leading to an IPSC. Compared with the only-PTB7-Th, the partial incorporation of Y6 serves a dual purpose: enhancing the NIR absorbance of the hybrid phototransistor and introducing additional trapping effects due to the discontinuous domain distribution for higher IPSC.

**
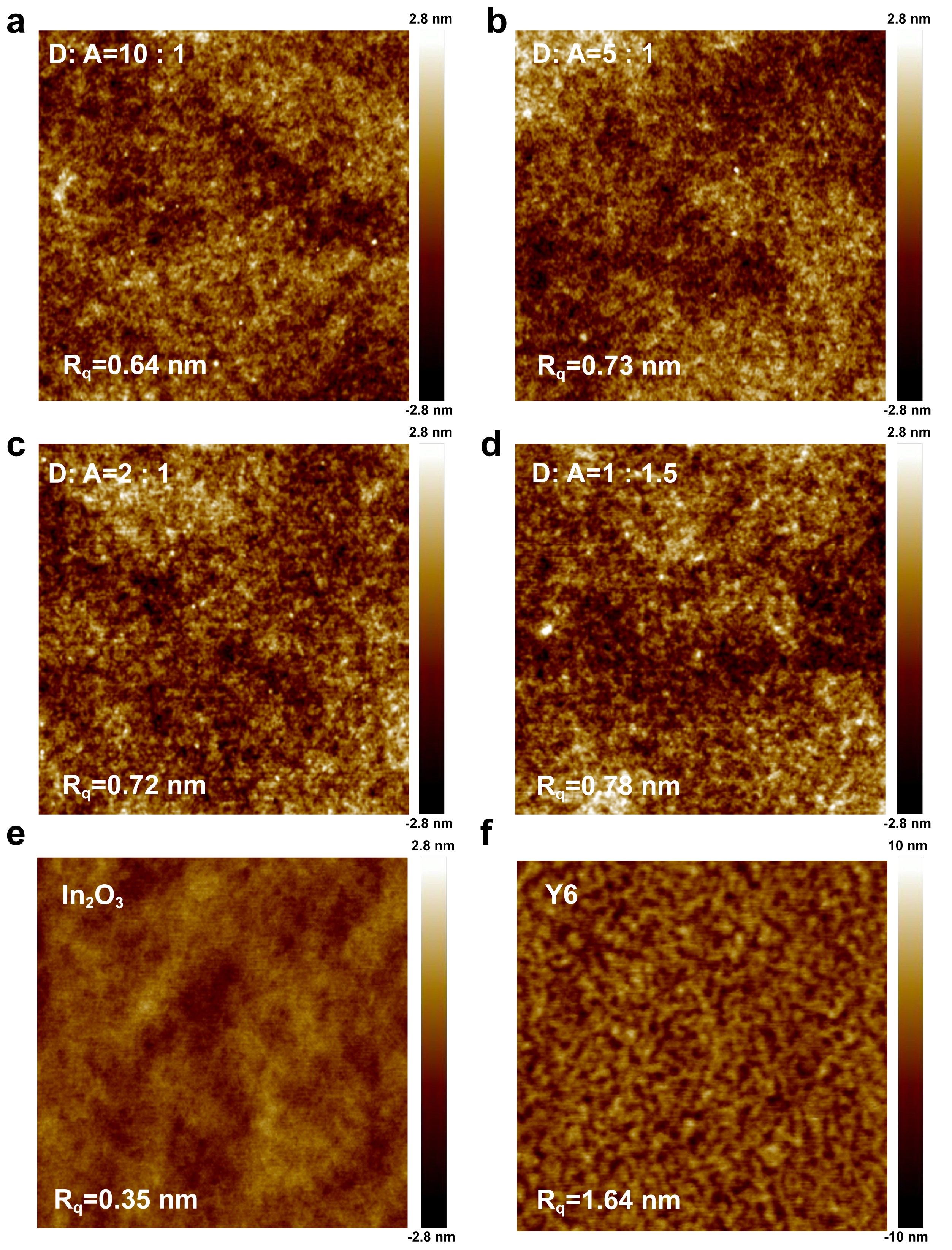
Figure S5.** **The surface morphologies of the organic and In_2_O_3_ films.** BHJ films all exhibited a comparable R_q_ value of approximately 0.7 nm. The In_2_O_3_ film displayed a smooth surface with a lower R_q_ value of 0.35 nm. The Y6 film, because of its greater crystalline structure, exhibited a rougher surface texture (R_q_ =1.64 nm). Each surface is with an area of 5 × 5 μm².


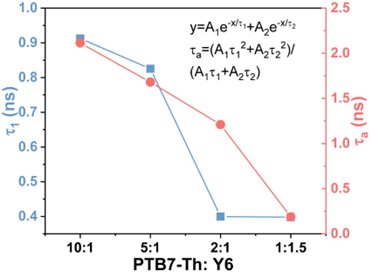


**Figure S6.** PL decay parameters τ_1_ and τ_a_ in different D/A ratio organic films.


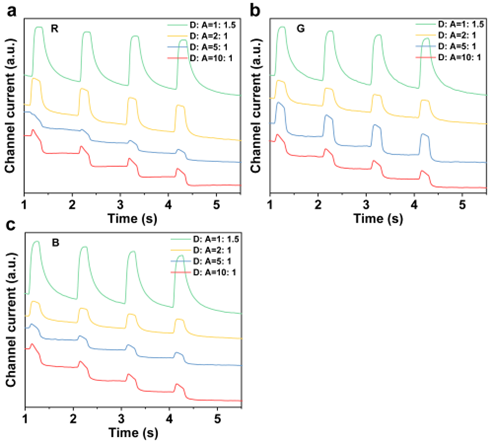


**Figure S7**. **The photoresponse of In_2_O_3_/BHJ phototransistors to visible light**. The hybrid phototransistor with varying D/A ratios of 1: 1.5, 2: 1, 5: 1, and 10: 1 under a sequence of four light pulses at different wavelengths: R (**a**), G (**b**), and B (**c**).


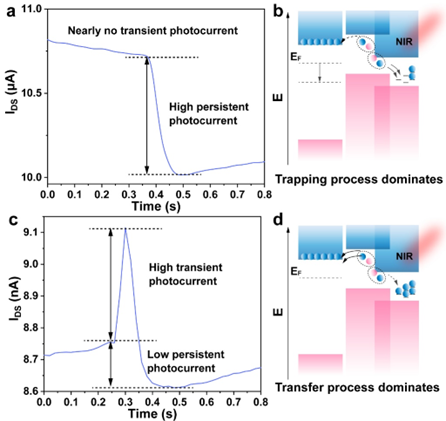


**Figure S8**. **The** **current change under the same NIR pulse stimulation with varying degrees of occupied trap sites in the BHJ**. (a, b) When trapping sites in the BHJ are nearly vacant and minimal electron accumulation occurs in the In2O3 layer, the device exhibits the highest IPSC, while the transient current diminishes. (c, d) When trapping sites are almost fully occupied, and electron accumulation in the In2O3 layer is significant, the device experiences the highest transient photocurrent. However, the IPSC is lower and decays faster.


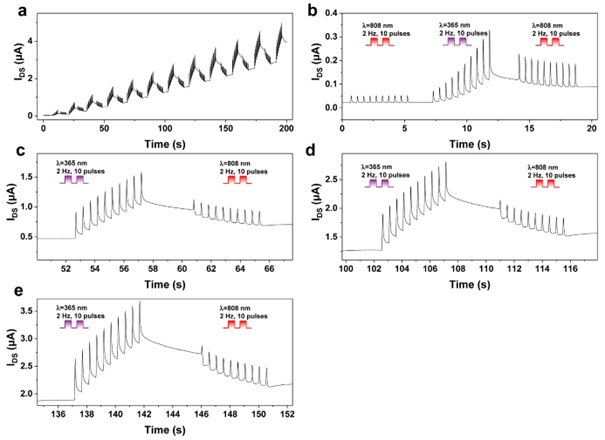
**Figure S9**. **The photoresponse under different baseline current.** (**a**) The dynamic photoresponse of the In_2_O_3_/BHJ phototransistor with a D/A ratio of 10: 1, was conducted through a series of cycles, each consisting of 10 pulses of UV and NIR light. The excitatory current was controlled to surpass the inhibitory current, thereby elevating the baseline, to acquire photoresponses at different base current levels. (**b**-**e**) The detailed examination focused on the photoresponse observed during cycle 1, cycle 4, and cycle 6 of the experimental sequence.


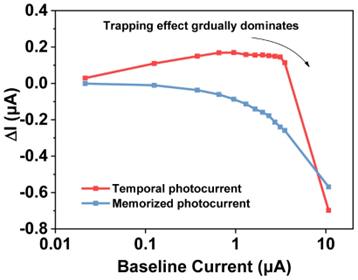


**Figure S10. The transient photoresponse and IPSC induced by NIR inhibitory stimulatio**n. It was observed that, with the escalation of the base current, the transient photocurrent exhibited an initial increase, followed by a gradual decrease, ultimately culminating in a reversal to a negative value. Concurrently, the absolute inhibitory current value demonstrated a gradual increase, signifying the increasing influence of electron trapping over the electron transfer process due to the accumulation electrons of In_2_O_3_ channel.


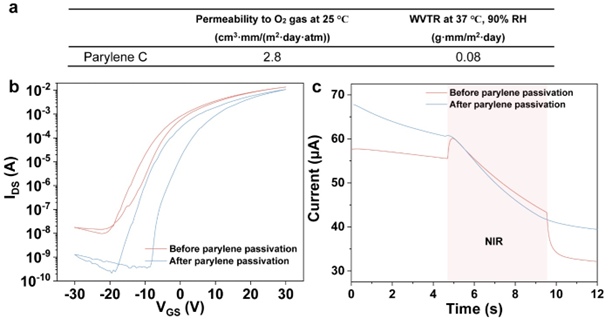
**Figure S11.** a) Parylene C water/oxygen permeability. b) Transfer curves before and after the parylene passivation (~1 μm). c) The channel under change under 5 s NIR irradiation before and after the parylene deposition at V_GS_=0 V V_DS_= 2 V.


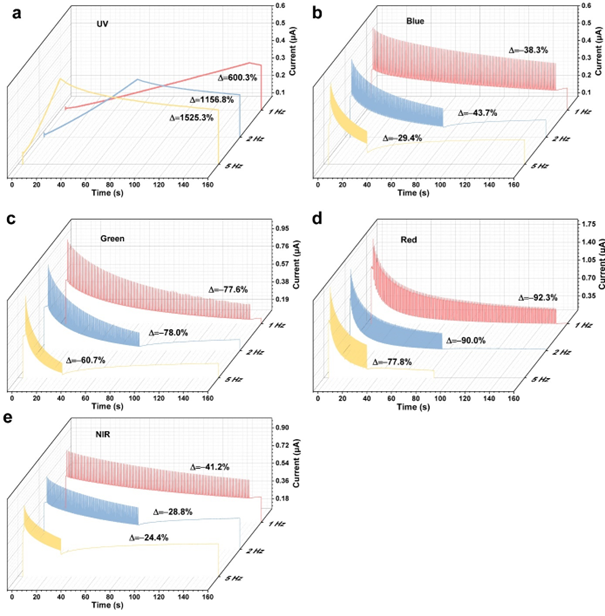
**Figure S12.** **The EPSC and IPSC characteristics of the hybrid phototransistor (D: A=10: 1) under various frequencies**. (**a**-**e**) The photoresponse under various wavelengths with each at frequencies of 1 Hz, 2 Hz, and 5 Hz (UV: 0.8 mW cm^-2^, R: 0.2 mW cm^-2^, G: 0.2 mW cm^-2^, B: 0.9 mW cm^-2^, and NIR: 3.1 mW cm^-2^). The illumination consisted of 150 light pulses, each with a pulse width of 100 ms. (V_GS_=0 V, V_DS_=0.5 V)


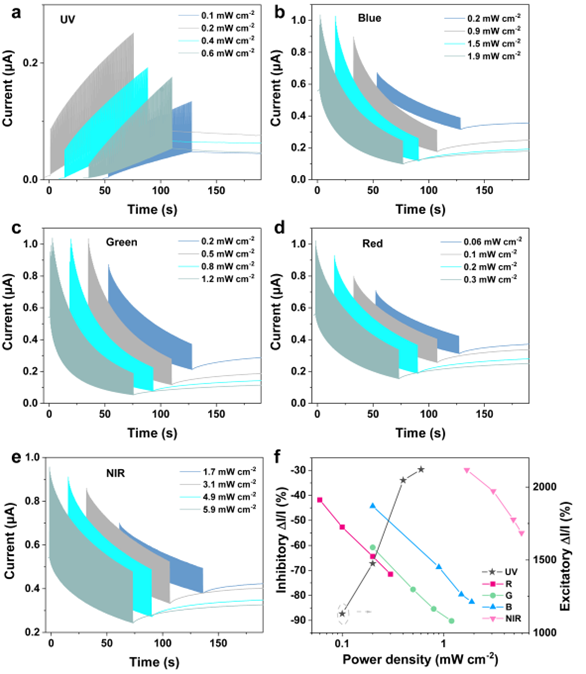
**Figure S13**. **The EPSC and IPSC characteristics of the hybrid phototransistor (D: A=10:1) under various power densities**. (**a**-**e**) The photoresponse of the hybrid phototransistor with a D/A ratio of 10: 1, at various power densities of UV, B, G, R, and NIR illumination, each at 2 Hz with 150 pulses and pulse width of 100 ms. (**f**) The relative change in base current extracted from measurements at various power densities, spanning from UV to NIR wavelengths. (V_GS_=0 V, V_DS_=0.5 V)


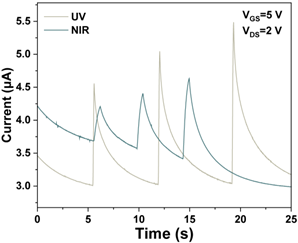


**Figure S14**. **The short-term plasticity under NIR and UV.** UV power density: 0.4, 0.5, 0.6 mW cm^-2^, NIR power density: 0.2, 0.3, 0.4 mW cm^-2^.


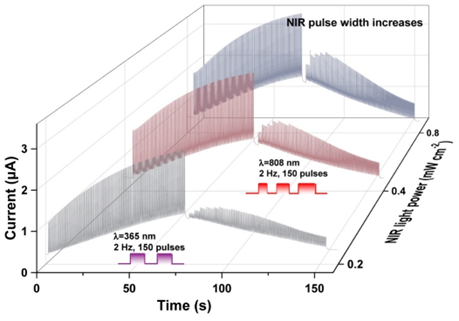


**Figure S15**. **The linearity modulation by extending the pulse width of NIR illuminatio**n. The UV light is set at a consistent power density and a fixed pulse width of 50 ms at 2 Hz. The pulse widths for the NIR illumination at the same 2 Hz frequency were systematically adjusted in the following manner: starting with the initial 40 ms pulse, the pulse width was systematically extended by an additional 20 ms after every sequence of 5 pulses. As a consequence, an almost linear inhibitory process was achieved across various power densities of NIR illumination (V_GS_= 0 V, V_DS_=2 V).


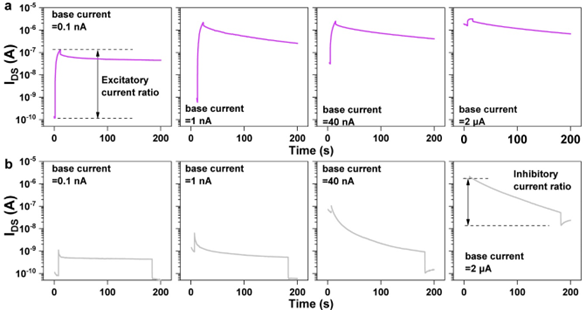
**Figure S16.** **Dynamic range at various baseline currents.** (**a**) The dynamic range during the excitatory process through the application of UV irradiation at various baseline current levels. (**b**) The dynamic range during the inhibitory process through the application of NIR irradiation at varying baseline current levels.


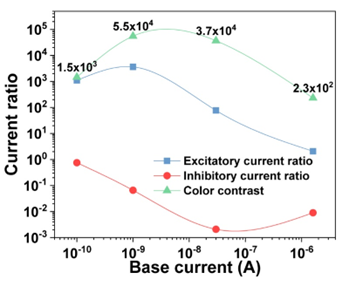


**Figure S17**. **The extracted overall dynamic range**. The dynamic range assessment of excitatory (UV irradiation) and inhibitory (NIR irradiation) processes, as well as the overall dynamic range, at varying levels of base current. (V_GS_=0 V, V_DS_=0.5 V)


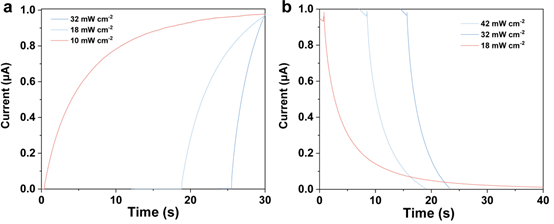


**Figure S18**. a) The excitation process under different power densities of the UV light. b) The inhibition process under different power densities of the NIR light.


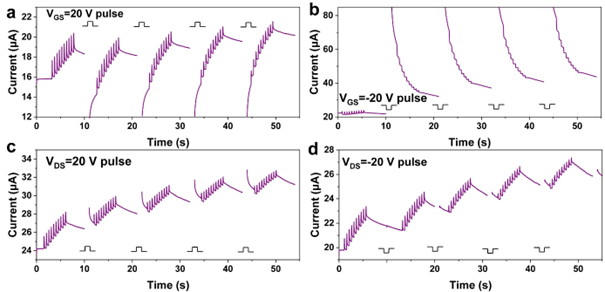
**Figure S19**. **The effect of V_GS_ and V_DS_ pulses on the excitatory process of the hybrid phototransistor** (UV light pulse frequency: 2 Hz, pulse width: 50 ms) (**a**) 10 UV light pulses were applied after each 1 s 20 V V_GS_ pulse. (**b**) 10 UV light pulses followed by each 1 s -20 V V_GS_ pulse. (**c**) 10 UV light pulses were applied following each 1 s 20 V V_DS_ pulse. (**d**) 10 UV light pulses were applied after each 1 s -20 V V_DS_ pulse.


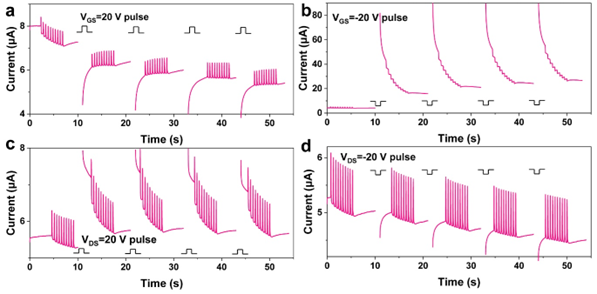
**Figure S20.** **The effect of V_GS_ and V_DS_ pulses on the inhibitory process of the hybrid phototransistor** (NIR light pulse frequency: 2 Hz, pulse width: 50 ms) (**a**) 10 NIR light pulses were applied after each 1 s 20 V V_GS_ pulse. (**b**) 10 NIR light pulses followed by each 1 s -20 V V_GS_ pulse. (**c**) 10 NIR light pulses were applied following each 1 s 20 V V_DS_ pulse. (**d**) 10 UV light pulses were applied after each 1 s -20 V V_DS_ pulse.


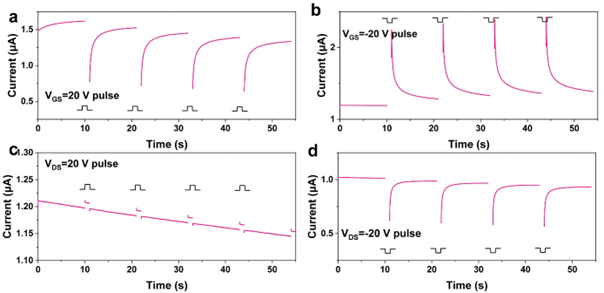
**Figure S21**. **The effect of V_GS_ and V_DS_ pulses on the reference In_2_O_3_ TFT.** (**a**) Four 20 V V_GS_ pulses (width: 1 s) were sequentially imposed. (**b**) Four -20 V V_GS_ pulses (width: 1 s) were sequentially imposed. (**c**) Four 20 V V_DS_ pulses (width: 1 s) were sequentially imposed. (**d**) Four -20 V V_DS_ pulses (width: 1 s) were sequentially imposed.


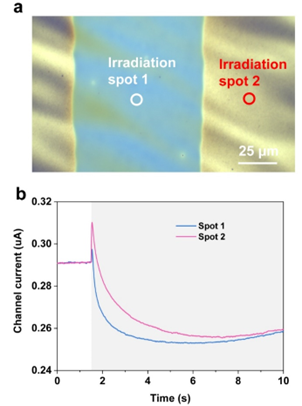


**Figure S22. The electrical field effect on the photoresponse.** A light spot of approximately 3 μm² of 530 nm from a Raman spectrometer was directed onto two distinct areas of the hybrid phototransistor (D: A=10: 1): the channel region (spot 1) and the electrode region (spot 2 under the conditions of _VGS_=0 V and V_DS_=5 V. Notably, the transient photocurrent was observed to be higher at spot 2 compared to spot 1. Conversely, the inhibitory current exhibited a reverse behavior. This phenomenon can be attributed to the fact that the vicinity of the electrode region features a higher electric field, facilitating efficient carrier extraction. In contrast, the channel region experiences a lower electric field, making carrier extraction less efficient and accentuating the trapping effect.


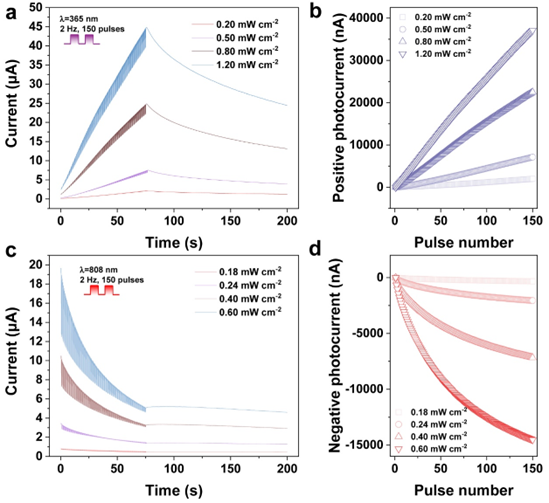


**Figure S23. High drain voltage synaptic performance.** The channel current of the hybrid phototransistor under 150 pulses of UV (**a**, **b**) and NIR (**c**, **d**) corresponding extracted photocurrent value at V_GS_=0 V, V_DS_=10 V.


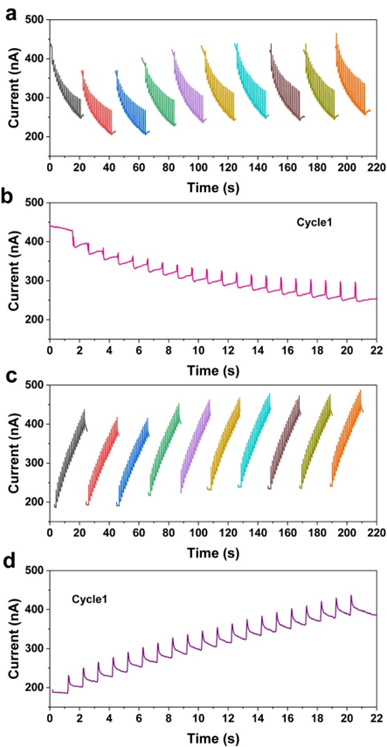


**Figure S24**. **Cycle-to-cycle variation.** (**a**) 10 cycles of the inhibitory process, each cycle consisting of 20 pulses of NIR irradiation at a frequency of 1 Hz, with each pulse having a duration of 100 ms. (**b**) Magnified current changes within the first cycle in (a). (**c**) 10 cycles of the excitatory process, with each cycle consisting of 20 UV pulses, also at a frequency of 1 Hz and with a pulse duration of 100 ms. (**d**) The magnified current changes during the initial cycle of the excitatory process, as described in (c).


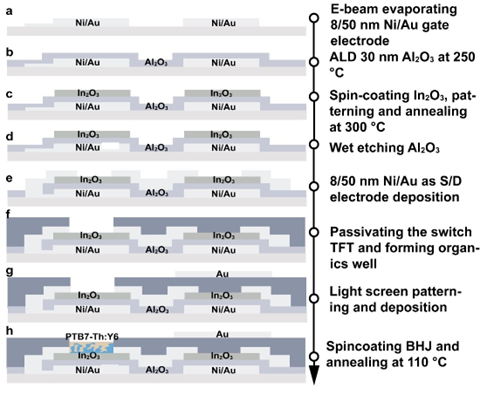


**Figure S25. The manufacturing process.** The schematic representation of the cross-section of the 1T1PT array.


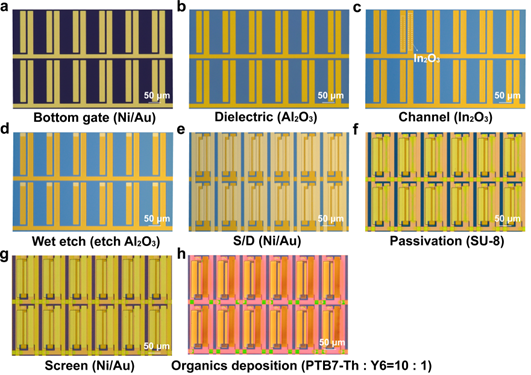


**Figure S26. The images at each fabrication step.** The optical images of the regional 1T1PT array at various stages of the fabrication process.


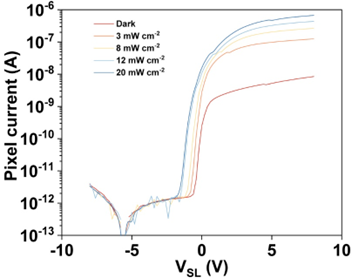


**Figure S27. The shield performance under UV irradiation.** The pixel current under various UV irradiation.


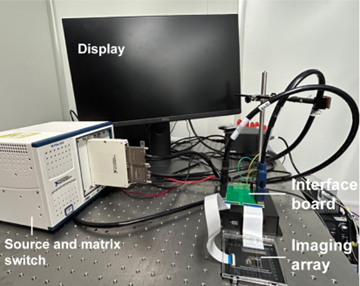


**Figure S28. Measurement setup for the dynamic imaging.**


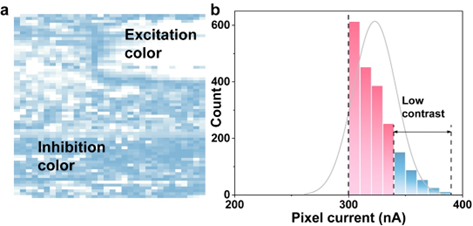


**Figure S29. The imaging results without spectral processing.** (**a**) The only positive response imaging with dual-band input. (**b**) The corresponding histogram exhibits low color contrast.


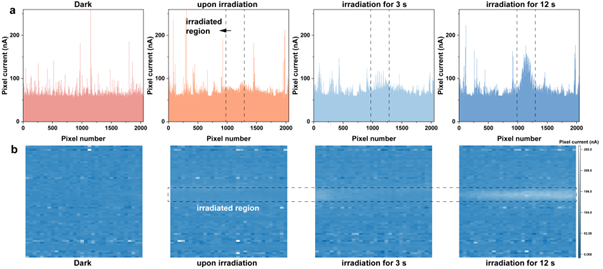
**Figure S30.** a) All the pixel current extracted from the active-matrix array under dark, upon irradiation, 3 s irradiation and 5 s irradiation at V_DL_=5 V, V_SL_=3 V. b) The corresponding imaging results to a).

**Table S1**. **The fitting parameters and averaged decay lifetimes of various organic films of different D: A ratios.**

| ***D: A*** | ***τ_1_*** | ***τ_2_*** | ***τ_a_*** | ***R^2^*** |
| --- | --- | --- | --- | --- |
| 10: 1 | 0.91248 | 4.23534 | 2.11 | 0.99674 |
| 5: 1 | 0.82525 | 4.7552 | 1.68 | 0.99499 |
| 2: 1 | 0.39961 | 3.00016 | 1.21 | 0.9806 |
| 1: 1.5 | 0.39795 | 0.4033 | 0.19 | 0.97485 |

**Table S2**. The comparison with recently reported optoelectronic device array.

| ***Structure*** | ***Array scale*** | | ***Pixel*** | ***Active-matrix*** | ***PPI*** | ***Wavelength (nm)*** | ***Dynamic range***  ***(dB)*** | ***Optical inhibition plasticity*** | ***Optical excitation plasticity*** | ***Trace imaging*** | ***Chromatic enhancement*** | ***Ref.*** |
| --- | --- | --- | --- | --- | --- | --- | --- | --- | --- | --- | --- | --- |
| In_2_O_3_/PTB7-Th: BTPV-4F | | 16×16 | 1T1PT | √ | 40 | 500-808 | - | × | × | × | × | ^[1]^ |
| IGZO/CdSe/CdSe/CdS | | 12×12 | 1T1PT | √ | ~42 | 406-638 | >150 | × | × | × | × | ^[2]^ |
| DNTT/BHJ | | 4×4 | 1PT1C | × | - | 416-784 | 103 | × | × | × | × | ^[3]^ |
| MoS_2_ | | 32×32 | 1PT | × | ~81 | 532 | ~60 | × | √ | × | × | ^[4]^ |
| BP | | 3×3 | 1PT | × | - | 1500-3000 | ~40 | × | √ | × | × | ^[5]^ |
| CNT/CsPbBr_3_ | | 16×16 | 1PT | × | - | 405, 516 | ~100 | × | √ | √ | × | ^[6]^ |
| BTBTT6-syn | | 6×8 | 1PT | × | - | 370-650 | ~55 | × | √ | √ | × | ^[7]^ |
| NbS_2_/MoS_2_ | | 10×10 | 1PT | × | - | 532 | ~12 | × | √ | √ | × | ^[8]^ |
| DTT-8/TFT-CN | | 5×5 | 1PT | × | - | 365 | 64 | × | √ | √ | × | ^[9]^ |
| MoS_2_ | | 20×20 | 1PT | × | - | 660 | - | × | √ | √ | × | ^[10]^ |
| PdSe_2_/MoTe_2_ | | 3×3 | 1PT | × | - | 365-980 | - | √ | √ | × | × | ^[11]^ |
| IGZO/CdS | | 7×7 | 1PT1T1R | × | ~23 | 525, 620 | ~40 | √ | √ | √ | × | ^[12]^ |
| BP/Al_2_O_3_/WSe_2_ | | 3×3 | 1PT | × | - | 450-637 | - | √ | √ | × | × | ^[13]^ |
| PbS/Gr/Pyr-GDY | | 7×6 | 1PT | × | - | 450, 980 | 10 | √ | √ | × | × | ^[14]^ |
| **In_2_O_3_/BHJ** | | **32×64** | **1TPT** | √ | **128** | **365-808** | **94** | **√** | **√** | **√** | **√** | **This work** |

BP: Black phosphorus, CNT: Carbon nanotube.

|  | **μ_sat_ (cm^-2^ V^-1^ s^-1^)** | **log(I_on_/I_off_)** | | **SS (mV dec^-1^)** | **V_th_ (V)** |
| --- | --- | --- | --- | --- | --- |
| Mean | 2.2 | 9.26 | 99.55 | | 0.911 |
| Standard Deviation | 0.11 | 0.34 | 8.43 | | 0.55 |
| Median | 2.28 | 9.31 | 101.04 | | 1.06 |

**Table S3**. The statistics for the pixel devices

**Supplementary Infomation References**

[1] D. Li, Z. Jia, Y. Tang, C. Song, K. Liang, H. Ren, F. Li, Y. Chen, Y. Wang, X. Lu, L. Meng, B. Zhu, *Nano Lett.* **2022**, *22*, 5434.

[2] J. Kim, C. Jo, M. G. Kim, G. S. Park, T. J. Marks, A. Facchetti, S. K. Park, *Adv. Mater.* **2021**, *34*, e2106215.

[3] A. Pierre, A. Gaikwad, A. C. Arias, *Nat. Photonics* **2017**, *11*, 193.

[4] H. Jang, C. Liu, H. Hinton, M. H. Lee, H. Kim, M. Seol, H. J. Shin, S. Park, D. Ham, *Adv. Mater.* **2020**, *32*, e2002431.

[5] S. Lee, R. Peng, C. Wu, M. Li, *Nat. Commun.* **2022**, *13*, 1485.

[6] Q. B. Zhu, B. Li, D. D. Yang, C. Liu, S. Feng, M. L. Chen, Y. Sun, Y. N. Tian, X. Su, X. M. Wang, S. Qiu, Q. W. Li, X. M. Li, H. B. Zeng, H. M. Cheng, D. M. Sun, *Nat. Commun.* **2021**, *12*, 1798.

[7] T. Jiang, Y. Wang, Y. Zheng, L. Wang, X. He, L. Li, Y. Deng, H. Dong, H. Tian, Y. Geng, L. Xie, Y. Lei, H. Ling, D. Ji, W. Hu, *Nat. Commun.* **2023**, *14*, 2281.

[8] P. Y. Huang, B. Y. Jiang, H. J. Chen, J. Y. Xu, K. Wang, C. Y. Zhu, X. Y. Hu, D. Li, L. Zhen, F. C. Zhou, J. K. Qin, C. Y. Xu, *Nat. Commun.* **2023**, *14*, 6736.

[9] X. Zhu, C. Gao, Y. Ren, X. Zhang, E. Li, C. Wang, F. Yang, J. Wu, W. Hu, H. Chen, *Adv. Mater.* **2023**, *35*, 2301468.

[10] J. Chen, Z. Zhou, B. J. Kim, Y. Zhou, Z. Wang, T. Wan, J. Yan, J. Kang, J. H. Ahn, Y. Chai, *Nat. Nanotechnol.* **2023**, *18*, 882.

[11] L. Pi, P. Wang, S.-J. Liang, P. Luo, H. Wang, D. Li, Z. Li, P. Chen, X. Zhou, F. Miao, T. Zhai, *Nat. Electron.* **2022**, *5*, 248.

[12] S. M. Kwon, J. Y. Kwak, S. Song, J. Kim, C. Jo, S. S. Cho, S. J. Nam, J. Kim, G. S. Park, Y. H. Kim, S. K. Park, *Adv. Mater.* **2021**, *33*, e2105017.

[13] Z. Zhang, S. Wang, C. Liu, R. Xie, W. Hu, P. Zhou, *Nat. Nanotechnol.* **2021**, *17*, 27.

[14] Y. X. Hou, Y. Li, Z. C. Zhang, J. Q. Li, D. H. Qi, X. D. Chen, J. J. Wang, B. W. Yao, M. X. Yu, T. B. Lu, J. Zhang, *ACS Nano* **2021**, *15*, 1497.
